# A Multi-scale Spatial Analysis of Native and Exotic Plant Species Richness Within a Mixed-Disturbance Oak Savanna Landscape

**DOI:** 10.1007/s00267-013-0120-y

**Published:** 2013-07-19

**Authors:** Timothy A. Schetter, Timothy L. Walters, Karen V. Root

**Affiliations:** 1 Metropolitan Park District of the Toledo Area, 5100 West Central Ave., Toledo, OH 43615 USA; 2 EnviroScience, Inc., Northwest Ohio Field Office, 6027 County Road 1, Swanton, OH 43558 USA; 3 Department of Biological Sciences, Bowling Green State University, Bowling Green, OH 43403 USA

**Keywords:** Exotic species, Landscape metrics, Midwest oak savanna, Plant species richness, Spatial heterogeneity, Spatial scale

## Abstract

Impacts of human land use pose an increasing threat to global biodiversity. Resource managers must respond rapidly to this threat by assessing existing natural areas and prioritizing conservation actions across multiple spatial scales. Plant species richness is a useful measure of biodiversity but typically can only be evaluated on small portions of a given landscape. Modeling relationships between spatial heterogeneity and species richness may allow conservation planners to make predictions of species richness patterns within unsampled areas. We utilized a combination of field data, remotely sensed data, and landscape pattern metrics to develop models of native and exotic plant species richness at two spatial extents (60- and 120-m windows) and at four ecological levels for northwestern Ohio’s Oak Openings region. Multiple regression models explained 37–77 % of the variation in plant species richness. These models consistently explained more variation in exotic richness than in native richness. Exotic richness was better explained at the 120-m extent while native richness was better explained at the 60-m extent. Land cover composition of the surrounding landscape was an important component of all models. We found that percentage of human-modified land cover (negatively correlated with native richness and positively correlated with exotic richness) was a particularly useful predictor of plant species richness and that human-caused disturbances exert a strong influence on species richness patterns within a mixed-disturbance oak savanna landscape. Our results emphasize the importance of using a multi-scale approach to examine the complex relationships between spatial heterogeneity and plant species richness.

## Introduction

Biodiversity is increasingly threatened by growing human impacts throughout the biosphere (Chapin and others 2000; Barnosky and others 2011). Mounting evidence suggests that loss of biodiversity may adversely affect ecosystem functioning (Hooper and others 2005; Cardinale and others 2006; Maestre and others 2012) along with key ecosystem services that provide for the well-being of humans on Earth such as climate regulation, water and air purification, soil fertility, erosion control, agricultural pest and disease control, and protection from natural hazards (Balvanera and others 2006; Diaz and others 2006; Mooney 2010). Faced with limited financial resources and a narrowing window of time to mitigate further loss of biodiversity, there is urgent need for resource managers to rapidly assess natural areas and prioritize various conservation actions across multiple scales, from individual sites to entire ecoregions (Novacek and Cleland 2001; Rey Benayas and others 2009).

Plant species richness (i.e., number of species) is frequently used to measure biodiversity (Cardinale and others 2011), ecosystem recovery (Ruiz-Jaen and Aide 2005), and ecological restoration (Wang 2010). Plant species richness is a logical choice as a monitoring and evaluation target for conservation because of the important functional role of plants as primary producers and as habitat for animal species (Cardinale and others 2011). Data on plant richness are relatively easy to collect and interpret compared to other formula-based diversity indices. In addition, it is useful to differentiate between species that are native to a given region and those that were introduced as a result of human actions (i.e., exotic species). Patterns in native and exotic richness may respond differently to various ecological processes (Denslow and others 2010). For example, in southern California shrublands, severe anthropogenic disturbances associated with urban and agricultural activities led to long-term reductions in native plant species richness and establishment of exotic annual grassland communities (Stylinski and Allen 1999). Thus, evaluation of native and exotic richness patterns in other native communities may provide useful information regarding specific ecological conditions.

Since it is usually possible to sample only a small fraction of a given landscape due to time and financial constraints, it is necessary to develop predictive models to provide information on native and exotic richness for the remaining unsampled landscape (Stohlgren and others 1997). Modeling relationships between richness and spatial heterogeneity (i.e., pattern) of biotic and abiotic resources across a given landscape offer a potentially useful approach. Spatial heterogeneity is hypothesized as one of the primary determinants of biodiversity (Huston 1994; Rosenzweig 1995), though the specific relationship between heterogeneity and diversity is often scale-dependent (Reed and others 1993; Tamme and others 2010). Recent studies evaluating a range of terrestrial ecosystems across multiple spatial scales have confirmed that relationships exist between plant species richness and various aspects of spatial heterogeneity, such as topography (Dogan and Dogan 2006; Dufour and others 2006; Thuiller and others 2006), landscape patch composition/configuration (Kumar and others 2006), soil depth (Lundholm and Larson 2003; Cingolani and others 2010), soil nutrients (Gilliam and Dick 2010), soil pH (Costanza and others 2011), water availability (Pausas and others 2003), grazing pressure (Olofsson and others 2008), and gradients in natural and human-caused disturbances (Deutschewitz and others 2003; Lilley and Vellend 2009).

To make better management and policy decisions to mitigate future loss of biodiversity, we require a better understanding of the connection between biodiversity and spatial heterogeneity at all scales so that we can make reliable predictions for scenarios of landscape change (Schröder and Seppelt  2006). Recent advances in the application of GIS and remote sensing technologies make these tools appealing for the rapid assessment of spatial heterogeneity and biodiversity (Luoto and others 2002). It is especially important to assess ecosystems or regions that contribute disproportionately to biodiversity (i.e., biodiversity hotspots) and those identified as critically endangered (Hoekstra and others 2005), such as the oak savanna region of the Midwestern United States.

Midwest oak savannas are among the most imperiled North American plant communities, having declined more than 99.9 % since European settlement due to land use change and fire exclusion (Nuzzo 1986; Noss and others 1995). Today, remnant oak savannas often represent local hotspots of biodiversity (Leach and Givnish 1999) and serve as refugia for rare species not found elsewhere on the landscape. Remnant oak savanna ecosystems are heavily influenced by mixed natural (fire and hydrologic cycles) and anthropogenic (land use conversion and habitat fragmentation) disturbances within the surrounding landscape (Grossmann and Mladenoff 2007). Studies of remnant oak savannas within a mixed-disturbance landscape have found relationships between plant richness and light availability (Leach and Givnish 1999), fire frequency (Weiher 2003; Peterson and Reich 2008), proximity to possible propagules (Brewer and Vankat 2006), intensity of restoration treatments (Abella and others 2001), and soil characteristics (Leach and Givnish 1999). Lilly and Velland (2009) evaluated relationships between spatial heterogeneity and plant species richness among remnant oak savannas in British Columbia, finding that gradients in human disturbance were important predictors of both native and exotic richness. However, relationships between spatial heterogeneity and plant richness remain largely unexplored for Midwest oak savannas.

The purpose of this study was to evaluate potential relationships between native/exotic plant species richness and spatial heterogeneity within the context of a mixed-disturbance oak savanna landscape. We followed the general approach offered by Kumar and others (2006), utilizing field data, remotely sensed data, and landscape pattern metrics to develop multi-scale predictive models of native and exotic plant species richness for remnant savanna, prairie and barrens communities. We chose to focus on these specific communities because they remain a target for ongoing conservation and restoration efforts throughout the Midwestern United States (Leach and Ross 1995; Abella and others 2007; Abella 2010). We examined the following specific research questions within the context of a mixed-disturbance oak savanna landscape: (1) Can we reliably predict native and exotic richness patterns using a subset of selected explanatory variables? (2) Do relationships between native/exotic richness and heterogeneity vary at different spatial scales? (3) Do these relationships vary within/among different plant community types?

## Study Area

**Fig. 1:**
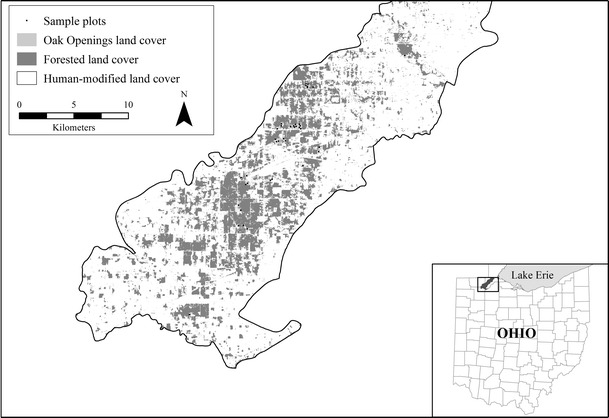
Map of study area (adapted from Schetter and Root 2011). Oak Openings land cover includes wet prairies, dry prairies, mesic prairies, sand barrens, and oak savannas. Human-modified land cover includes urban/residential lands, croplands, conifer plantings, Eurasian meadows, and artificial ponds

The 478 km^2^ Oak Openings region of northwestern Ohio (41°25′ to 41°44′N; 83°34′ to 84°2′W) occurs near the eastern extent of the historic Midwest Oak Savanna region (Nuzzo 1986). The region’s climate is humid continental; mean monthly temperatures range from −10 to 23 °C; mean annual precipitation is 81 cm (USDA–NRCS 2010). Historically, the region featured a mosaic of oak savanna uplands and wet prairie lowlands occurring on postglacial sandy soils (Brewer and Vankat 2004). Following European settlement, the region was systematically altered through drainage, fire exclusion, urban development, and row-crop agriculture. Today, roughly 73 % of the region has been converted to human-modified land cover types while less than 3 % of the region remains covered by native savannas, prairies and barrens; now heavily fragmented and imbedded within a matrix of human-modified and forested land cover types (Fig. 1, Schetter and Root 2011). Despite these changes, the region continues to harbor one-third of Ohio’s state-listed rare plant and animal species within an area that collectively represents less than 0.5 % of Ohio’s total land area. Currently, 10 % of the region’s total land area has been permanently protected as public parks and nature preserves by various conservation organizations including the Metropolitan Park District of the Toledo Area, Ohio Department of Natural Resources, and The Nature Conservancy. Although human-caused disturbances persist throughout much of the region, the Oak Openings’ remaining natural areas continue to be influenced by natural disturbances such as seasonal flooding and prescribed fires set by preserve managers. Refer to Schetter (2012) for more detailed descriptions of the region’s plant communities and their current conservation status.

## Methods

### Ecological Classification Hierarchy and Land Cover Map

**Fig. 2:**
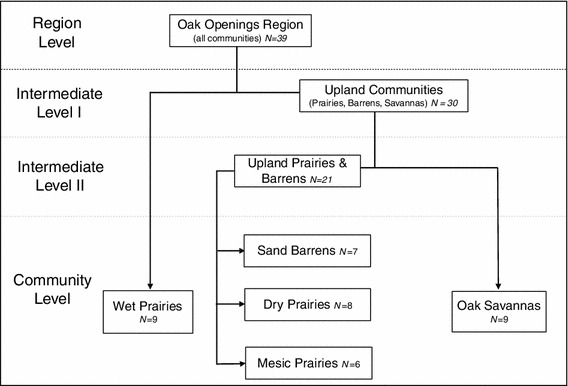
Five Oak Openings plant communities within the context of an ecologically based vegetation classification hierarchy developed for the Oak Openings region of northwestern Ohio

We used an ecologically based vegetation classification hierarchy to evaluate relationships between species richness and spatial heterogeneity among the study area’s native Oak Openings communities (Fig. 2). At the broadest level within the hierarchy, “Region Level,” study sites representing all native Oak Openings communities were evaluated. At the first intermediate level, all upland community types were evaluated separately from wetland communities. At the second intermediate level, upland communities were further divided into prairies/barrens or savannas. At the finest level within this hierarchy, five discrete Oak Openings community types were evaluated, including wet prairies, mesic prairies, dry prairies, sand barrens, and oak savannas. These Oak Openings communities were mapped, along with forested and human-modified land cover types using 30-m pixel Landsat satellite image (Schetter and Root 2011, Fig. 1). The resulting raster land cover map of our study area represented 15 total land cover classes.

### Site Selection and Field Sampling

Using the land cover map of our study area (Schetter and Root 2011) imported into ArcGIS 9.1 (ESRI, Redlands, CA, USA), we randomly sampled 30-m map pixels stratified by five community types, resulting in 39 total study sites (Fig. 2). At each study site, we established a 20 × 50 m (1,000-m^2^) modified-Whittaker, multi-scale plot (Kalkhan and Stohlgren 2000) with the long axis randomly assigned to either a north–south or east–west bearing. Plots were centered within two adjacent 30-m map pixels and located on the ground using a high-precision GPS unit (Trimble GPS Pathfinder Pro XRS) set to NAD83 Ohio State Plane North coordinate system. Minimum distance between plots was 100 m. We excluded potential plot locations consisting of mixed community types or those intersected by human features such as roads or ditches. For each study site we noted whether it was located within an existing managed preserve. Based on a review of site management histories, we were able to determine that all study sites occurring within managed preserves received multiple restoration treatments over several years (e.g., prescribed burning, mowing, and spot spraying of herbicide to control invasive species). However, lack of detailed management histories for most of these sites prevented us from further evaluating the potential effects of specific restoration treatments.

Each modified-Whittaker plot included 10 1-m^2^ non-overlapping subplots, two 10-m^2^ non-overlapping subplots, and one 100-m^2^ subplot, each nested within the 1000-m^2^ plot. Within each 1-m^2^ subplot, we estimated foliar cover for each vascular plant species at ground level (<1.7 m height) to the nearest 1 %, along with bare ground, litter (attached), duff (detached), coarse woody debris, cryptobiotics (mosses, algae, and lichens), and tree/shrub canopy (>1.7 m). Cover for species occupying <1 % of a 1-m^2^ subplot was recorded as 0.5 %. Due to layering of foliage, litter, duff, and cryptobiotics, it was possible for cumulative cover to exceed 100 %. We recorded cumulative number of plant species within each of the 10-m^2^ subplots, the 100-m^2^ subplot, and the 1,000-m^2^ plot. Within each 1,000-m^2^ plot, we recorded by species all woody stems ≥2.5 cm dbh (diameter at breast height). All upland communities were sampled from 26 July to 20 September 2008 and from 2 August to 22 September 2009, corresponding to peak biomass for these communities. For wet prairies, sampling occurred from 23 May to 2 July 2009, coinciding with availability of flowers and fruits within the Family *Cyperaceae* (necessary for their successful identification) rather than onset of peak biomass within these sedge-dominated communities. Therefore estimates of cover could not be directly compared between upland and wet prairie communities. For all communities, species were classified as either native or exotic to our study area following Andreas and others (2004). Species were identified following Voss (1972, 1985, 2004). Plant species not identified in the field were collected for comparison with appropriate taxonomic keys and herbarium specimens.

Within each modified-Whittaker plot, we collected five soil samples (one from each corner and one from the plot center) to a depth of 40 cm using a 2.5-cm diameter soil probe after removing any surface litter. For each plot, soil samples were pooled into a single sample following Kumar and others (2006) and air dried for 48 h. Pooled samples were submitted to a commercial analytical lab (Brookside Laboratories, Inc., New Knoxville, OH, USA) where they were ground to pass through a 2-mm sieve. Soil texture (sand, silt, and clay fractions) was determined following the standard hydrometer method (ASTM 2002). Soils were analyzed for total nitrogen, total carbon, and organic carbon following Nelson and Sommers (1996). Extractable calcium, magnesium, potassium, sodium, and sulfur were determined following Suarez (1996).

### GIS Data Collection

To evaluate the relationship between specific environmental gradients and plant species richness, we measured proximity of each 1,000-m^2^ plot to nearest patch edge, paved roadway, water source (dug pond or drainage ditch), and human dwelling using high-resolution color orthophotos of our study area (OGRIP 2006; Lucas County ARIES 2004) imported into ArcGIS 9.1. We selected proximity to patch edge as a variable of interest because patch edges are known to influence plant dispersal patterns (Fagan and others 1999). The other variables were selected to evaluate gradients in anthropogenic disturbance. Proximity to natural streams was initially considered as a variable of interest but was later dismissed because no natural surface water drainage occurred within 0.5 km of any of our research plots. We evaluated topographic heterogeneity within and among research plots using 0.762-m grid digital elevation model (DEM) data of our study area (OGRIP 2006). We extracted DEM data for each 1,000-m^2^ plot (approx. 1,700 data points per plot) and measured the following variables using ArcGIS 9.1 Spatial Analyst (ESRI, Redlands, CA, USA): mean elevation (m), slope (%), and aspect (radians) transformed into north–south and east–west gradients (see Kumar and others 2006). We used within-plot standard deviation of elevation to quantify topographic variability following Dufour and others (2006).

### Landscape Pattern Analysis

We evaluated landscape heterogeneity at each study site by measuring selected landscape pattern metrics at two nested spatial extents (60-m and 120-m) surrounding each 1,000-m^2^ study plot. Using program FRAGSTATS, version 3.3 (McGarigal and Marks 1995), we performed moving window analyses using both 60-m and 120-m circular windows around each research plot (corresponding to an area of 1.89 and 6.21 ha, respectively, see McGarigal and Marks 1995). The raster land cover map of the region was used as the basis for all analyses (Schetter and Root 2011; ESRI GRID format, NAD 1983 datum, Ohio State Plane North projection, 30-m pixel size). We did not use spatial extents greater than 120-m due to overlap of landscape windows among several research plots at larger spatial extents. The 8-cell patch neighbor rule was applied to all analyses (i.e., cells of the same land cover type were considered part of the same patch if they touched either orthogonally or diagonally). We used five commonly used landscape pattern metrics (calculated in FRAGSTATS at the landscape level) to quantify specific aspects of landscape composition/configuration (see Li and Reynolds 1994):

- Cohesion Index: measures physical connectedness of patches on the landscape
- Landscape Shape Index: measures total patch edge adjusted for landscape size (edge density)
- Patch Richness Density: measures number of different patch types present per total landscape area
- Shannon’s Diversity Index: measures the proportional abundance of each patch type on the landscape
- Percentage of Landscape: measures total area of all patches of the corresponding patch type per total landscape area

### Statistical analyses

Our general statistical approach was to test for linear relationships between native or exotic plant species richness (response variables) and selected physical/landscape variables (potential predictor variables) at each level within the Oak Openings region ecological classification hierarchy (Fig. 1) and then develop a “best” predictive model among all significant predictor variables for native and exotic richness at each of these levels using multiple linear regression techniques following Kumar and others (2006). All statistical analyses were performed using JMP ver. 9.0 (SAS Institute, Inc.) unless otherwise referenced. First, as a variable screening step, we conducted univariate linear regression at each ecological level to remove potential predictor variables that were not significantly related to native/exotic richness at each ecological level using a critical value of *P* = 0.05. For all variables, we tested for normality within the residuals using the Shapiro–Wilks test and examined residual plots for obvious patterns indicative of heteroscedasticity. Data were transformed when appropriate prior to analysis to reduce the influence of non-normality/heteroscedasticity within the datasets (e.g., arcsine square root transformation for percent data, log_10_ (*N* + 1) transformation for count data). Data exhibiting strong non-linear relationships following transformation were excluded from linear regression analyses.

To account for spatial autocorrelation within the linear regression models, we followed the procedure developed by Dutilleul (1993) using a computer program written by Legendre (2000). This procedure provides an estimate of the degrees of freedom lost due to spatial dependence between *x* and *y* variables, giving a corrected *F* value and corresponding *P* value for each linear regression model (Dale and Fortin 2002). Potential predictor variables not significant at *P* < 0.05 after correcting for spatial autocorrelation were eliminated from further consideration. Remaining predictor variables were further evaluated using stepwise forward multiple regression (*P* = 0.25 to enter model, *P* = 0.10 to leave model) to develop a set of candidate models of native/exotic richness at both 60-m and 120-m spatial extents within each of the four levels of the Oak Openings ecological hierarchy. Before conducting multiple regression analyses, we examined all predictor variables for cross-correlations and multicollinearity by evaluating correlation matrices and inverse correlation matrices of each set of predictor variables. Any variables with cross correlations >±0.75 or those with variance inflation factors >2.5 were not included in the same model (Neter and others 1996; Kumar and others 2006). Following this variable screening step, at the three highest ecological levels between three and four potential predictor variables were entered into each native richness model, while between five and seven potential predictor variables were entered into each exotic richness model. At the community level, we were unable to develop multiple regression models after completing the variable screening process.

At the three highest ecological levels, we used Akaike’s Information Criteria adjusted for small sample size (AICc) to select the “best” model among all possible candidate models for native and exotic richness at both 60-m and 120-m spatial extents. Only candidate models with Δ AICc of ≤2 were given consideration (Burnham and Anderson 2002). In cases where multiple candidate models had Δ AICc of ≤2, the model with the fewest variables was selected as the most parsimonious model. For all multiple regression models, we assumed a multivariate normal distribution with constant variance in the residuals and no spatial autocorrelation.

## Results

### Plant Species Richness Among Oak Openings Communities

**Fig. 3:**
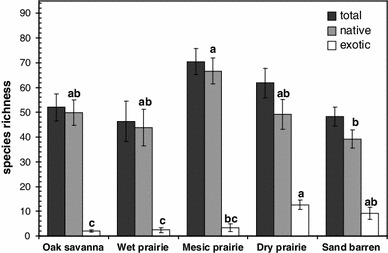
Mean plant species richness (per 1,000 m^2^ plot) among five Oak Openings communities for all, native, and exotic species. *Error bars* are one standard deviation. Means without *shared letters* (comparing total, native, and exotic species richness across community type) differ at *P* < 0.05 (Tukey’s test)

Among five Oak Opening community types, we recorded 406 vascular plant species (349 native, 57 exotic), including 48 species listed as endangered, threatened, or potentially threatened in Ohio (ODNR 2010). This accounted for one-third of the region’s known vascular plant flora (Moseley 1928; Walters 2007) and 34 % of the region’s documented state-listed rare plant species within a sampled area of 3.9 ha (<0.01 % of the Oak Openings region’s total land area). Less than 2 % of specimens observed in the field could not be positively identified to species. Refer to Schetter (2012) for a complete list of recorded species. Total species richness was not significantly different among community types (Fig. 3). Native richness tended to be greatest in mesic prairies while it tended to be lowest in sand barrens. Exotic richness was four to six times greater in dry prairies and sand barrens compared to the other community types. Native richness was positively correlated with exotic richness only among wet prairies (*R* ^2^ = 0.74, *F*_1,7_ = 9.72, *P* = 0.044, corrected for spatial autocorrelation following Dutilleul (1993)). Among the upland communities and at the three higher levels of the classification hierarchy, there was no statistically significant relationship between native and exotic richness (*P* < 0.05). Thirty out of 39 research plots occurred within existing managed preserves including all oak savanna, mesic prairie and wet prairie plots; while four of eight dry prairie plots and two of seven sand barrens plots occurred within managed preserves. While the effects of specific management and restoration treatments could not be evaluated from our data, the fact that oak savanna, mesic prairie, and wet prairie sample sites were not found outside of existing managed preserves within our sampling design supports existing evidence that large-scale intact remnants of these communities do not persist in the Oak Openings region without regular ecological management treatments such as prescribed fire (see Schetter and Root 2011).

### Relationships Between Spatial Heterogeneity and Species Richness

At the region level (among all study sites), there were no significant relationships between native richness and the physical/landscape variables that we measured (*P* < 0.05; Table 1), which we attribute to differences in community composition and underlying site conditions (e.g., soils and hydrology) between the sedge-dominated wet prairie communities and the upland prairie/savanna communities. At the region level, individual physical/landscape variables explained 8–46 % of the observed variation in exotic richness (Table 2). Among all upland communities (first intermediate level), individual physical/landscape variables explained 10–52 % of observed variation in native richness (Table 1) and 11–58 % of exotic richness (Table 2). Among upland prairies and barrens (second intermediate level), explanatory power of measured variables generally improved for both native and exotic richness (20–50 % and 15–61 %, respectively). At these three ecological levels, landscape variables at the 60-m extent consistently explained more variation in native richness compared to the 120-m extent, while landscape variables at the 120-m extent consistently explained more variation in exotic richness than at the 60-m extent.

**Table 1:** Relationship between native species richness and individual predictor variables at three levels of an ecologically based vegetation classification hierarchy

Variable type	Predictor variable	Adj.* R* ^2^	Coeff.	Modified^a^		
				df	*F*	*P*
Entire region (*n* = 39)	No variables significant at *P* < 0.05					
Uplands (*n* = 30)						
Physical	Slope (%)	0.23	−0.033	27.1	9.60	0.004
	*C*_total_ (%)	0.11	0.103	29.0	4.62	0.040
	*C*_organic_ (%)	0.13	0.111	29.0	5.52	0.026
	Clay (%)	0.10	0.028	29.0	4.25	0.049
Landscape (60-m extent)	Oak Openings land cover (%)^b^	0.52	0.007	13.2	15.3	0.002
	Human-modified land cover (%)^c^	0.32	−0.006	15.3	8.02	0.012
Landscape (120-m extent)	Oak Openings land cover (%)^b^	0.21	0.003	20.0	6.20	0.022
	Human-modified land cover (%)^c^	0.18	−0.004	20.0	5.41	0.031
Upland prairies and barrens (*n* = 21)						
Physical	Total foliar cover (%)	0.40	0.003	16.5	12.5	0.003
	Total ground litter (%)	0.24	0.003	20.0	7.46	0.013
	Bare ground (%)	0.42	−0.005	17.2	13.7	0.002
	Topographic variability (m)	0.34	−0.676	20.0	11.1	0.003
	Slope (%)	0.38	−1.379	17.7	12.6	0.002
Landscape (60-m extent)	Oak Openings land cover (%)^b^	0.48	0.006	7.5	11.0	0.012
	Human-modified land cover (%)^c^	0.50	−0.007	10.0	11.3	0.007
Landscape (120-m extent)	Savanna land cover (%)	0.20	0.005	15.2	4.88	0.043
	Human-modified land cover (%)^c^	0.27	0.012	12.5	5.37	0.038

Only variables significant at *P* < 0.05 are shown. A complete list of variables evaluated is provided by Schetter (2012)

^a^Native species richness was log_10_ (*N* + 1) transformed prior to analysis. Values for df, F, and P were adjusted for spatial autocorrelation following Dutilleul (1993)

^b^Composite of all five Oak Openings land cover classes (wet prairie, mesic prairie, dry prairie, sand barren, and oak savanna)

^c^Composite of Eurasian meadow, perennial ponds, dense urban, residential/mixed, turf/pasture, cropland, and conifer plantation land cover types (see Schetter and Root 2011)

**Table 2:** Relationship between exotic species richness and individual predictor variables at three levels of an ecologically based vegetation classification hierarchy

Variable type	Predictor variable	Adj.* R* ^2^	Coeff.	Modified^a^		
				df	*F*	*P*
Entire region (*n* = 39)						
Physical	Total ground litter (%)	0.24	−0.007	36.3	12.91	0.001
	Bare ground (%)	0.10	0.008	37.0	5.17	0.029
Landscape (60-m extent)	Shannon Diversity Index	0.08	0.320	38.0	4.34	0.046
	Eurasian meadow land cover (%)	0.23	0.021	28.7	9.73	0.004
	Human-modified land cover (%)^c^	0.22	0.014	32.5	10.37	0.003
Landscape (120-m extent)	Landscape Shape Index	0.12	0.333	38.0	6.25	0.017
	Patch richness density	0.08	0.003	37.5	4.30	0.045
	Shannon Diversity Index	0.11	0.308	38.0	5.46	0.025
	Savanna land cover (%)	0.24	−0.008	25.4	8.76	0.007
	Eurasian meadow land cover (%)	0.46	0.034	22.4	20.02	<0.001
	Oak Openings land cover (%)^b^	0.12	−0.007	30.9	5.31	0.028
	Human-modified land cover (%)^c^	0.31	0.014	37.0	16.80	<0.001
Uplands (*n* = 30)						
Physical	Total foliar cover (%)	0.27	−0.007	29.0	11.78	0.002
	Distance from roads (m)	0.19	−0.001	17.3	4.84	0.042
	Distance from water (m)	0.29	−0.001	29.0	12.64	0.001
Landscape (60-m extent)	Patch richness density	0.13	0.002	24.3	4.77	0.039
	Eurasian meadow land cover (%)	0.11	0.013	26.3	6.40	0.018
	Human-modified land cover (%)^c^	0.31	0.016	15.5	7.77	0.014
Landscape (120-m extent)	Cohesion Index	0.20	−0.017	24.3	4.77	0.039
	Eurasian meadow land cover (%)	0.38	0.030	20.9	13.82	0.001
	Savanna land cover (%)	0.58	−0.011	12.0	17.48	0.001
	Human-modified land cover (%)^c^	0.43	0.015	15.6	12.79	0.003
Upland prairies and barrens (*n* = 21)						
Physical	Total foliar cover (%)	0.16	−0.005	20.0	4.95	0.039
	Soil Na	0.24	0.016	17.1	6.59	0.020
	Soil S	0.23	−0.022	20.0	6.85	0.017
Landscape (60-m extent)	Oak Openings land cover (%)^b^	0.25	−0.011	14.8	5.77	0.030
	Human-modified land cover (%)^c^	0.24	0.012	14.0	5.47	0.035
Landscape (120-m extent)	Cohesion Index	0.25	−0.017	20.0	7.74	0.012
	Landscape Shape Index	0.26	0.428	20.0	7.96	0.011
	Patch richness density	0.15	0.003	20.0	4.47	0.048
	Savanna land cover (%)	0.61	−0.018	13.8	23.16	<0.001
	Eurasian meadow land cover (%)	0.37	0.028	19.0	12.31	0.003
	Oak Openings land cover (%)^b^	0.38	−0.010	14.9	10.78	0.005
	Human-modified land cover (%)^c^	0.43	0.012	15.0	12.42	0.003

Only variables significant at *P* < 0.05 are shown. A complete list of variables evaluated is provided by Schetter (2012)

^a^Exotic species richness was log_10_ (*N* + 1) transformed prior to analysis. Values for df, F, and P were adjusted for spatial autocorrelation following Dutilleul (1993)

^b^Composite of five Oak Openings land cover classes (wet prairie, mesic prairie, dry prairie, sand barren, and savanna)

^c^Composite of Eurasian meadow, perennial ponds, dense urban, residential/mixed, turf/pasture, cropland, and conifer plantation land cover types (see Schetter and Root 2011)

At the three highest ecological levels, native and exotic richness showed contrasting relationships with various measures of spatial heterogeneity (Tables 1, 2). For example, for native species richness we found positive correlations with measures of within-plot vegetative cover and percent Oak Openings land cover surrounding plots while we observed negative relationships between native richness and measures of within-plot topographic heterogeneity, landscape heterogeneity surrounding plots, and percent human-modified land cover surrounding plots. In contrast, we found negative relationships between exotic richness and measures of vegetative cover and percent Oak Openings land cover while we observed positive relationships between exotic richness and measures of landscape heterogeneity and percent human-modified land cover. Within individual community types, there were fewer physical/landscape predictor variables that were statistically significant compared to the higher ecological levels, which we attributed at least in part to small sample sizes at the community level. Individual variables at the community level explained 60–89 % and 44–66 % of variability in native and exotic richness, respectively (Table 3). A summary of all physical and landscape attributes that we evaluated is available online (Schetter 2012, pp. 55–57).

**Table 3:** Relationship between native/exotic species richness and individual predictor variables within five Oak Openings plant communities

	Species richness^a^	Variable type	Predictor variable	Adj.* R* ^2^	Coeff.	Modified^a^		
						df	*F*	*P*
Oak Savanna (*n* = 9)	Native	Landscape (60-m extent)	Upland forest land cover (%)	0.72	−0.009	6.5	19.97	0.004
	Exotic	Landscape (120-m extent)	Upland prairie land cover (%)	0.45	0.013	7.8	8.34	0.022
Wet Prairie (*n* = 9)	Native	Physical	Total foliar cover (%)	0.60	0.018	4.2	7.98	0.045
	Exotic	Physical	Total litter (%)	0.52	−0.010	5.1	6.96	0.045
		Landscape (120-m extent)	Human-modified land cover (%)^b^	0.65	0.021	4.3	9.98	0.032
Mesic Prairie (*n* = 6)	Native		No variables significant (*P* < 0.05)					
	Exotic		No variables significant (*P* < 0.05)					
Dry Prairie (*n* = 8)	Native		No variables significant (*P* < 0.05)					
	Exotic	Landscape (60-m extent)	Oak Openings land cover (%)^c^	0.44	−0.010	6.1	6.64	0.044
		Landscape (120-m extent)	Oak Openings land cover (%)^c^	0.60	−0.007	5.0	9.65	0.028
Sand Barren (*n* = 7)	Native	Physical	Slope (%)	0.77	−0.041	4.5	19.50	0.009
		Physical	Bare ground (%)	0.89	−0.004	3.2	30.61	0.010
	Exotic	Physical	Proximity to water (m)	0.66	−0.003	3.6	9.16	0.047

Only variables significant at *P* < 0.05 are shown. A complete list of variables evaluated is provided by Schetter (2012)

^a^Species richness was log_10_ (*N* + 1) transformed prior to analysis. Values for df, F, and P were adjusted for spatial autocorrelation following Dutilleul (1993)

^b^Composite of Eurasian meadow, perennial ponds, dense urban, residential/mixed, turf/pasture, cropland, and conifer plantation land cover types (see Schetter and Root 2011)

^c^Composite of five Oak Openings land cover classes (wet prairie, mesic prairie, dry prairie, sand barren, and savanna)

### Best Explanatory Models of Native and Exotic Richness

At the three highest ecological levels a single “best” multiple regression model was developed separately for native and exotic richness (Tables 4 and 5, respectively), explaining 50–69 % of the variation observed within our data. At these three ecological levels, models of exotic richness consistently explained more variation in our data than models of native richness. A model for native richness could not be developed at the region level due to lack of statistical significance of individual predictor variables. At both intermediate levels, the best models of native richness included landscape variables at the 60-m scale. Best models of exotic richness at the region and intermediate levels included landscape variables at the 120-m scale. At the individual community level, multiple regression models of native and exotic richness could not be developed because of the small number of variables that were statistically significant after adjusting for spatial autocorrelation and/or high levels of cross-correlation when more than one variable was significant.

**Table 4:** Best models of native plant species richness at three levels of ecological hierarchy

Spatial extent	Native species richness predictor variable^a^	Parameter estimate	*P*	Adjusted *R* ^2^	AICc	Δ AICc
Entire region (*n* = 37)^b^						
60-m	No variables significant at *P* < 0.05					
120-m	No variables significant at *P* < 0.05					
Uplands (*n* = 29)^c^						
60-m	**Clay soil (%)**	**0**.**018**	<**0**.**0001**	**0**.**56**	−**44**.**21**	**0**
	**Oak Openings land cover (%)** ^d^	**0**.**006**				
120-m	Slope (%)	−0.021	0.002	0.37	−32.29	11.93
	Clay soil (%)	0.023				
	Oak Openings land cover (%)	0.002				
Upland prairies and barrens (*n* = 20)^c^						
60-m	**Human-modified land cover** **(%)** ^e^	−**0**.**007**	**0**.**007**	**0**.**50**	−**26**.**12**	**0**
120-m	Bare ground (%)	−0.004	0.003	0.49	−21.59	3.65
	Human-modified land cover (%)^e^	−0.0001				
	Savanna land cover (%)	0.004				

The best model at each ecological level is shown in bold type

^a^Native species richness was log_10_ (*N* + 1) transformed prior to analysis

^b^Sample size was reduced by two due to missing data

^c^Sample size was reduced by one due to missing data

^d^Composite of all five Oak Openings land cover classes (wet prairie, mesic prairie, dry prairie, sand barren, and oak savanna)

^e^Composite of Eurasian meadow, perennial ponds, dense urban, residential/mixed, turf/pasture, cropland, and conifer plantation land cover types (see Schetter and Root 2011)

**Table 5:** Best models of exotic plant species richness at three levels of ecological hierarchy

Spatial extent	Exotic species richness predictor variable^a^	Parameter estimate	Adjusted			
			*P*	*R* ^2^	AICc	Δ AICc
Entire region (*n* = 37)^b^						
60-m	Total ground litter (%)	0.003	<0.0001	0.56	15.34	9.43
	Upland prairies & barrens land cover (%)	0.008				
	Human-modified land cover (%)^c^	0.017				
120-m	**Total ground litter (%)**	**0**.**002**	<**0**.**0001**	**0**.**62**	**6**.**59**	**0**.**68**
	**Upland prairies & barrens land cover (%)**	**0**.**010**				
	**Human-modified land cover (%)** ^c^	**0**.**017**				
Uplands (*n* = 29)^d^						
60-m	Total foliar cover (%)	−0.003	<0.0001	0.60	14.80	12.17
	Distance from roads	−0.001				
	Distance from water	−0.001				
	Human-modified land cover (%)^c^	0.007				
	Upland prairies & barrens land cover (%)	0.003				
120-m	**Total foliar cover (%)**	−**0**.**323**	<**0**.**0001**	**0**.**69**	**3**.**32**	**0**.**70**
	**Distance from roads**	−**0**.**001**				
	**Savanna land cover (%)**	−**0**.**009**				
Upland prairies and barrens (*n* = 20)^d^						
60-m	Soil Na (ppm)	0.016	<0.0001	0.65	4.24	8.59
	Soil S (ppm)	−0.028				
	Oak Openings land cover (%)^e^	−0.003				
120-m	**Soil Na (ppm)**	**0**.**132**	<**0**.**0001**	**0**.**77**	−**4**.**35**	**0**.**00**
	**Soil S (ppm)**	−**0**.**016**				
	**Savanna land cover (%)**	−**0**.**011**				

The best model at each ecological level is shown in bold type

^a^Exotic species richness was log_10_ (*N* + 1) transformed prior to analysis

^b^Sample size was reduced by two due to missing data

^c^Composite of Eurasian meadow, perennial ponds, dense urban, residential/mixed, turf/pasture, cropland, and conifer plantation land cover types (see Schetter and Root 2011)

^d^Sample size was reduced by one due to missing data

^e^Composite of all five Oak Openings land cover classes (wet prairie, mesic prairie, dry prairie, sand barren, and oak savanna)

## Discussion

Within the context of a mixed-disturbance oak savanna landscape, our results showed three consistent trends in the relationship between plant species richness and spatial heterogeneity. First, we found that multiple regression models of species richness consistently explained more variation for exotic species than for native species, supporting the findings of Kumar and others (2006) that exotic plant species are more sensitive to spatial heterogeneity than native plant species. Second, among all measures of spatial heterogeneity that we evaluated, we found that in most cases landscape composition derived from raster land cover data explained more variation in our data than other possible explanatory variables. Specifically, we found that percentage of human-modified land cover within the surrounding landscape was negatively correlated with native species richness but positively correlated with exotic species richness. Third, we found that exotic richness was better explained at a larger spatial extent (roughly 6 ha) surrounding research plots while native richness was better explained at a smaller spatial extent (roughly 2 ha) surrounding research plots. These findings, which were generally consistent across all levels of our ecological classification hierarchy, point to the strong influence of landscape-scale human disturbances on species richness in our study area and also highlight potential differences in adaptive strategies between native and exotic species in response to these disturbances.

The profound negative effects of human-caused habitat loss on species richness are well documented (Fahrig 2003). However, native and exotic species often respond differently to landscape-scale habitat fragmentation associated with habitat loss. Within fragmented landscapes, exotic plant species are often found in greater abundance along habitat patch edges (McDonald and Urban 2006) and along road corridors (Jodoin and others 2008; Lilly and Velland 2009) compared to native species. In addition, increased anthropogenic disturbances associated with habitat fragmentation such as drainage alterations, livestock grazing, and soil disturbance have been found to negatively impact native plant species richness while leading to increases in exotic plant species richness (McIntyre and Lavorel 1994; Honnay and others 1999). While individual life-history traits of native and exotic species vary widely (Sutherland 2004), it is likely that these human disturbances, along with intentional introduction of exotic species (through sources such as agriculture/horticulture) lead to greater exotic species propagule pressure; while greater density of roads and habitat edges provide corridors for their direct dispersal and facilitate easier movement of their potential vectors (Lilly and Velland 2009). It is also likely that these same factors have negative impacts on many native species through direct habitat loss and by creating barriers to their dispersal between habitat patches. This scenario would explain our observation that exotic species richness exhibited stronger relationships with spatial heterogeneity at a broader spatial extent compared with native species richness.

Within the heavily fragmented Oak Openings region, we found ample evidence of the influence of human-caused disturbances associated with habitat fragmentation. Among landscape variables, we found that exotic species richness was positively correlated with amount of patch edge (measured by Landscape Shape Index), relative number of patches on the landscape (measured by Patch Richness Density and Shannon Diversity Index), proximity to roads, and proximity to man-made ditches/ponds; but was negatively correlated with patch connectedness (measured by Cohesion Index). Among soil nutrients, we observed a positive correlation between native richness and soil organic carbon among upland sites, which may be related to the well-established effects of soil disturbance on reducing soil organic carbon (e.g., Post and Kwon 2000). In addition, levels of soil sodium among upland prairies and barrens were positively correlated with exotic richness but also positively correlated with proximity to roads (*R* = 0.56, *P* < 0.01), a likely source of soil sodium through runoff of road salt.

In contrast to studies of plant species richness in mountainous regions linking species richness and gradients in elevation (Dogan and Dogan 2006; Kumar and others 2006), we found no such relationship within the relatively flat Oak Openings region. However, for upland communities (especially sand barrens) we found a negative relationship between native species richness and measures of within-plot topographic heterogeneity. Other regional-scale studies have shown the importance of topographic heterogeneity in explaining plant species richness (Dufour and others 2006; Thuiller and others 2006; Costanza and others 2011). However these studies have shown positive relationships between species richness and heterogeneity. Our contrasting results again point to the strong influence of human disturbances within our study area. Although broad-scale topography within the Oak Openings can be attributed to glacial and post-glacial natural processes (Forsyth 1970), we found that increased site-level topographic heterogeneity within our study can be attributed to more recent human disturbances. For example, we found the greatest site-level topographic heterogeneity among sand barrens communities. A quick review of available USGS topographic maps and aerial photos of our study sites revealed that all of the sand barrens we evaluated originated from human disturbances since the mid-twentieth century (e.g. sand pits, former homesteads, and off-road vehicle use).

We acknowledge that our findings are based on a single observation of each of our research plots and that the correlations we observed do not necessarily point to causal relationships between heterogeneity and richness. We also note that although our study area is referred to as the Oak Openings “region”, the land area under investigation in our study was on the order of several hundred square kilometers in contrast to other larger “regions”, for example the Midwestern United States. We do not discount the importance of other factors known to influence the relationship between heterogeneity and plant species richness, such as climate, geology, and natural disturbances (e.g., fire regime and hydrologic cycles) which were not evaluated in our study.

### Landscape Composition as a Rapid Assessment Tool

Previous studies have established a clear justification for using plant species richness as a basis for measuring ecosystem restoration success, both theoretically (Wang 2010) and in practice (Ruiz-Jaen and Aide 2005). However, it is usually not practical to measure species richness across an entire area of interest, especially at larger spatial scales. Therefore it is critical for effective regional conservation planning that appropriate surrogates are developed to quantify patterns of plant species richness (Ferrier 2002). Much of the physical data we collected in the field (such as vegetative cover and soil characteristics) have been shown to reliably predict plant species richness across multiple spatial scales and ecosystems. However, these data can be time-consuming and costly to collect. Therefore it is especially appealing to find appropriate surrogates of plant species richness through remote sensing and GIS applications for rapidly assessing a given area for conservation planning. For the Oak Openings region, we found percentage of human-modified land cover in the landscape to be especially promising in this regard. Percentage of landscape has been used to reliably predict wetland condition (Mack 2006) and is currently used by regulatory agencies as part of a rapid assessment method for wetlands (Mack 2001). Based on our results, percentage of landscape should be given strong consideration as a rapid assessment tool for predicting plant species richness across mixed-disturbance landscapes.
